# Working in hospitals during a pandemic: investigating the resilience among medical staff during COVID-19 outbreak through qualitative and quantitative research

**DOI:** 10.1017/S1463423622000305

**Published:** 2022-09-07

**Authors:** Zahra Hatefipour, Zahra Maghami Sharif, Hojjatollah Farahani, Asma Aghebati

**Affiliations:** 1 Faculty of Education Sciences and Psychology, Ferdowsi University, Mashhad, Iran; 2 Faculty of Humanities and Social Sciences, Science and Research Branch, Islamic Azad University, Tehran, Iran; 3 Assistant Professor, Department of Psychology, Tarbiat Modares University, Tehran, Iran; 4 Assistant Professor, Clinical Psychology Department, School of Behavioral Sciences and Mental Health, Tehran Institute of Psychiatry, Iran University of Medical Sciences, Tehran, Iran

**Keywords:** ARM-R, coronavirus, COVID-19, employee stress, medical staff, resilience

## Abstract

**Background:**

Medical staff in hospitals were faced with great stress as a result of COVID-19’s sudden and severe occurrence, which makes investigating their resilience essential.

**Aims and methods:**

Using qualitative and quantitative research methods, this research studied medical staff (*n* = 403) working in a hospital during the COVID-19 pandemic and followed four main goals: First was evaluating the psychometric properties of the Persian version of Adult Resilience Measure-Revised (ARM-R). The second goal was investigating the personal, relational, social, and organizational issues facing the medical staff during the COVID-19 using semi-structural interviews. The third goal was to determine predictive effects of demographic and work-related variables on resilience using stepwise regression analysis. And the fourth was comparing resilience of three groups of the medical staff (coronavirus group consisted of the medical staff in direct contact with COVID-19 patients; emergency group who work in the emergency department who deal with both COVID and non-COVID patients; and non-coronavirus group who had no contact with COVID-19 patients) using one-way ANOVA.

**Findings:**

Results showed that internal reliability/consistency, content, and face validity of the Persian version of the ARM-R are acceptable. The construct validity of the test was also verified using exploratory factor analysis and indicated the two factors of personal and relational resilience. The content of the interviews was analyzed using manifest content analysis, and the results were divided into 27 subcategories and 3 main categories including personal, organizational, and family categories. Moreover, regression analysis revealed that the marital status and age of children can explain resilience variance in some medical staff groups. The results of ANOVA and post hoc test also showed that the total resilience of the non-coronavirus group was greater than the coronavirus and emergency groups; the relational resilience of the coronavirus and non-coronavirus groups was greater than the emergency, and non-coronavirus group’s personal resilience was greater than the emergency group.

## Introduction

Many studies showed that medical staff experiences pressure and stress because of the demanding nature of their role and the environmental elements in which they operate (Beh and Loo, 2012; Foureur *et al.*, 2013; Dyrbye *et al*., 2014; Liang *et al.*, 2018; Grace and VanHeuvelen, 2019). They also confront serious mental health issues like depression, anxiety, and symptoms of post-traumatic stress (PTSD) (Ahmed *et al.*, 2009; Mealer *et al*., 2012; Pipe *et al*., 2012; Koinis *et al.*, 2015; Grace and VanHeuvelen, 2019). The sudden COVID-19 outbreak in late 2019, which was declared a pandemic soon after, further added to the complexities. The COVID-19 pandemic is a global-scale health crisis (Wang *et al.*, 2020) and the most critical since the severe acute respiratory syndrome epidemic back in 2003 believed to have much more devastating effects (Hawryluck *et al*., 2004). At the time of the present study, the last report of the World Health Organization (WHO) emphasized that COVID-19 had already affected more than 45 million individuals around the world, causing over 1 million deaths (WHO, 2020). With the increasing number of infected cases and deaths, hospitals were in more demand than ever. As a result, medical staff was facing a heavy workload, more significant stressors, risks, and more challenges in coping with the crisis (Petzold *et al.*, 2020). Some of them might experience avoidance by their family or community owing to stigma or fear. These circumstances made an already challenging situation far more difficult (World Health Organization, 2020) and could affect their mental health as well (Huang *et al*., 2020; Liang *et al*., 2020; Lu *et al.*, 2020). Lu *et al.* (2020) found that during the COVID-19 epidemic, anxiety and stress disorder incidence are high among China’s medical staff. In their study, the anxiety incidence and the scores of PTSD in female medical staff were higher than that in the male, and the anxiety incidence in nurses was higher than in doctors. Xiao *et al.’s* research showed that the sleep quality of medical staff who treated COVID-19 was relatively low because doctors and nurses had to wear protective clothing every day, including hazardous materials suits, HazMat, and the team worked continuously in the isolation wards with high work intensity and under pressure (Xiao *et al.*, 2020). Research about vicarious traumatization among nurses who work with COVID-19 patients revealed that the vicarious traumatization scores for frontline nurses were significantly lower than those of non-front-line nurses (Li *et al.*, 2020). Greenberg also asserts that in dealing with challenges of the COVID-19 pandemic, healthcare staff is at the risk of moral injury and mental health problems (Greenberg *et al*., 2020).

Resilience is an important factor that enables people to thrive in the face of crisis (Davydov *et al*., 2010). Individuals’ capacity to navigate their way to the psychological, social, cultural, and physical resources that sustain their well-being, as well as their ability to negotiate with others for these resources to be provided in culturally meaningful ways, is what resilience means when facing significant adversity (Ungar, 2013). Previous research showed the importance of resiliency in medical staff’s personal and professional life and their mental health. Tubbert showed that resiliency played an active role in the lives of emergency nurses. They shared stories about their work experience as a nurse and demonstrated how resiliency skills have a positive influence on their personal and professional lives by providing skills to handle stress and acuity inherent to work in the emergency department (Tubbert, 2016). Pipe *et al.* (2012) observed that resiliency is correlated to positive strategies for coping and enhancing the well-being of the healthcare workers, personally and organizationally. In Mealer *et al.* (2012) study, researching ICU nurses showed that the presence of resilience was significantly associated with a lower prevalence of PTSD, psychological symptoms of burnout syndrome, as well as symptoms of depression and anxiety. Dalia *et al.* (2013) also found that higher resilience levels in medical staff were significantly associated with lower PTSD symptoms and emotional exhaustion. Moreover, Manzano García and Ayala Calvo (2012) stated that resilience is a protective factor against emotional exhaustion for nurses and suggested that workplace environments should be designed to promote their resilience. Besides, in Dyrbye study (2010), compared to vulnerable medical students, resilient medical students were less likely to experience depression, report higher levels of social support, perceive learning climate as more positive, and experience less stress and fatigue.

These findings provided an enhanced appreciation and awareness of resiliency within the medical staff. Since the COVID-19 crisis causes much pressure on them and affects their mental health, researching resiliency is becoming more essential. The present research uses quantitative and qualitative methods to achieve the following four goals: The first was evaluating the psychometric properties of the Persian version of Adult Resilience Measure (ARM-R) for the first time. The 28-item version of this measure has been adequately studied, but to the best knowledge of the authors, no study investigated the psychometric properties of ARM-R with 17 items in any language other than English. We chose this scale because of its special properties. ARM-R is designed for assessing only adults’ resiliency (18 years old and older). It is a culture-independence scale that can assess resilience in different socio-cultural contexts. ARM-R has simplified wording with 3- and 5-point response scales to help researchers choose the appropriate form depending on the participants. Although there is a version of the ARM with 28 items, it has been emphasized by Michael Ungar, founder and director of the Resilience Research Centre (RRC), Dalhousie University, Canada, to use the 17-item measure for scientific research because it is simpler and shorter. Studying this measure, therefore, is of high importance (RRC, 2022).

The second goal was identifying the main problems facing the medical staff members using qualitative research methods. The staff members’ personal, social, relational, and organizational issues were investigated through interviews and evaluated by manifest content analysis.

The third goal was to determine if the demographic and work-related variables can predict the resilience in different groups of medical staff or not, and the fourth aim was comparing three groups of the medical staff using quantitative research methods. The first group, named coronavirus, consisted of the medical staff in direct contact with COVID-19 patients; the second group, named the emergency, consisted of the medical staff working in the emergency department who deals with both COVID and non-COVID patients, and the third group, named non-coronavirus, consisted of the staff who had no contact with COVID-19 patients. It was assumed that frequent contact with COVID-19 patients and the subsequent risk factors result in lower resilience. Therefore, the two first groups (coronavirus and emergency) would have lower resilience scores. No similar research on resilience has been conducted based on the comparison of medical staff in separate groups.

## Methods

### Ethical consideration

Before conducting the study, the research proposal was endorsed by the ethics committee of Mashhad University of Medical Sciences. After developing the manuscript, the final version was fully approved.

### Participants

This study was cross-sectional, and participants were selected using convenience sampling from the medical staff (including physicians, residents, stagers, nurses, attendants, psychologists, and crew) of 3 departments including the coronavirus department, emergency center, and non-coronavirus departments of oncology, hemato- and rheumatology, burns unit, gastroenterology, nephrology, emergency poisoning, and internal ward in the Imam Reza Hospital in Mashhad, Iran. The study was conducted during the peak of the coronavirus epidemic in Iran from February 20 to April 19, 2020. According to Kline (2000), the adequate number of variables in each group is one hundred. Based on this number, the sample sizes were set as follows: 131 of the medical staff working with Corona patients were placed in group 1, ‘the coronavirus group’; 131 were selected from the emergency staff and placed in group 2, ‘the emergency group’; and 141 were selected from the other staff members and placed in group 3, ‘the non-coronavirus group’. A total of 403 members participated in the study, and Table 1 shows their demographic information.

**Table 1.** Demographic information of participants

		Coronavirus		Non-coronavirus		Emergency		Missing	Total
Characteristics		Frequency	Percent	Frequency	Percent	Frequency	Percent		
Participants		131	32.5%	141	35.5%	131	32.5%	–	403
Gender	Male	49	12.25%	47	11.75%	53	13.25%	3	400
	Female	81	20.25%	93	23.25%	77	19.25%		
Education	Diploma and under Diploma	28	7%	29	7.25%	23	5.75%	3	400
	University Education	78	19.5%	97	24.25%	77	19.25%		
	Medical student	22	5.5%	9	2.25%	26	6.5%		
	Attendant	3	0.75%	3	0.75%	5	1.25%		
Marital status	Single	46	11.47%	22	5.48%	34	8.47%	2	401
	Married	85	21.19%	117	29.17%	97	24.18%		

### Measurement

ARM-R (RRC, 2022) is self-report measures of social-ecological resilience and is used by researchers and practitioners worldwide. It was developed from the perspective that resilience is a social-ecological construct. This is a revised version of the Adult Resilience Measure and is suitable for adults aged 18 or older. The ARM-R has 17 items with scores from 3- or 5-point Likert scales. All items in the measures are positively worded which simplifies scoring by easily summing up the scores. The items can be directly summed to gain an individual’s resilience total score (minimum = 17, maximum = 85). Also, two subscales can be computed: personal resilience and relational resilience. Relational resilience involves the important relationships with either a primary caregiver, a partner, or the family, and its characteristics. Intrapersonal and interpersonal items are categorized under personal resilience. The two subscales are interconnected in that they rely on the social ecologies of an individual to reinforce their resilience. High scores indicate resilience-related characteristics for both the overall measure and subscales.

### Translation of ARM-R

#### Forward translation

With official permission from the original questionnaire developer, Professor Michael Ungar, two bilingual translators translated the questionnaire into their mother tongue(Farsi) as it is highly recommended in the literature that two independent translators perform the first-time translations from the original language separately (Guillemin *et al*., 1993; Beaton *et al*., 2007). One of them was aware of the questionnaire concepts and provided a translation that more closely resembles the original instrument. The other one was a translator who was unaware of the objective of the questionnaire and produced the second translation in order to detect the subtle differences in the original questionnaire. Discrepancies between translators were discussed and resolved by an unbiased, bilingual translator who was not involved in the previous translations.

#### Backward translation

As with the forward translation, the backward translation (Persian to English) was performed by two other translators to ensure the accuracy of the translation. To avoid bias, back-translators did not become aware of the intended concepts of the questionnaire measures. Original questionnaire developer, Ungar, checked, and approved the forward and backward translations.

#### Preliminary pilot testing

Preparing the pre-final version of the translated questionnaire should undergo pilot testing on a small sample (about 30–50) before the real test (Guillemin *et al.*, 1993; Beaton *et al*., 2007). The 30 participants were randomly chosen from the medical staff and were asked to answer the translated questionnaire. Then, respondents were asked (verbally by an interviewer) to elaborate on what they thought each questionnaire item and their corresponding response meant, how did they choose their answers, how much the options represent the concept that exists in their mind, and which words or sentences are unclear. Finally, the authors revised the questionnaire based on the feedback. The researchers could then assure of the relative synonymity of translated items with the original items and the absence of any confusion as a result of translation.

### Statistical methods

In this study, four main goals were pursued, and different procedures and analyses were used to examine each of them. SPSS software version 26 was utilized for statistical analysis at alpha = 0.5. Missing data were below 5%, which was replaced by means.

The first aim was assessing the psychometric properties of the ARM-R which was followed by using explanatory factor analysis and Cronbach’s alpha correlation to measure construct validity and internal reliability, respectively. We used manifest content analysis in order to analyze interviews related to our second goal, which was examining the issues that medical staff faced during the pandemic. The Pearson correlation and stepwise regression analysis were applied to predict resilience by demographic and work-related variables related to our third goal. Regarding the fourth aim, one-way ANOVA was used to compare the mean resilience in three separate groups of medical staff, and then, Scheffé post hoc test was used to compare the means one by one.

## Results

Participants’ gender, marital status, and education frequencies of participants reported by groups are presented in Table 1. The mean age of participants was 34 years, the mean number of their children was one, and the average age of these children was 6 years.

### Psychometric properties of ARM-R

#### Construct validity: factor analysis of ARM-R

The structure of ARM-R was evaluated using an explanatory factor analysis of the 403 participants. Table 2 shows the Kaiser-Meyer-Olkin (KMO) and Bartlett’s test results. The KMO Measure of sampling adequacy is a statistic concerned with the proportion of variance in the variables that might be caused by underlying factors. KMO values higher than 0.8 indicate that the sample is adequate (Tabachnick *et al*., 2007). Our research has adequate KMO = 0.863.

**Table 2.** KMO and Bartlett’s test of ARM-R structure

KMO		.863
Bartlett’s Test of Sphericity	Approx. Chi-Square	1891.104
	df	120
	Sig.	.000

KMO = Kaiser-Meyer-Olkin measure of sampling adequacy.

The equivalence of the correlation matrix with an identity matrix is a hypothesis investigated through Bartlett’s test of sphericity. This test can determine whether the study variables are related or not and therefore unsuitable for structure detection. Small values (less than 0.05) of the significance level indicate that factor analysis may be useful with study data (Tabachnick *et al.*, 2007). Bartlett’s test results in our research were significant and showed the factorization of the tool’s items.

As most of the questionnaire items had an abnormal distribution, principal axis factoring was used for the explanatory factor analysis. Also, as the correlation coefficient of factors was less than 0.3, a varimax rotation was used to reach the simple structure. The two factors collectively saturated the tool and indicated 57.460 of the variance. The inclusion criterion for factors was an eigenvalue over 1. Table 3 shows the results of the exploratory factor analysis of ARM-R, and Table 4 presents the factor loadings after rotation.

**Table 3.** Exploratory factor analysis of ARM-R

Factor	Initial eigenvalues	% of Variance	Cumulative %
1	3.26	30.397	30.397
2	2.7	27.063	57.460

**Table 4.** Rotated factor matrix^a^ of ARM-R

Items (Questions)	Factor	
	1	2
Q1		.559
Q2		.607
Q3		.525
Q4	.621	
Q5	.625	
Q6		.384
Q7		.550
Q8	.631	
Q9	.399	.469
Q10	.356	.393
Q11	.766	
Q12	.387	.387
Q14		.540
Q15	.710	
Q16		.523
Q17	.619	

Extraction method: principal axis factoring.

Rotation method: varimax with Kaiser normalization.

a Rotation converged in 3 iterations.

Factor loading results indicated a loading under 0.3 for item 13. This item was therefore eliminated as the minimum loading, according to Tabachnick *et al*. (2007), was over 0.33. The inclusion and exclusion criteria of items were as follows: a minimum of 0.33 of factor loading; no cross-loadings with a difference under 0.1, and a communality greater than 0.4. Consequently, the (two-) factor structure of the resilience questionnaire was verified by eliminating an item. In the Persian version, item 6, ‘If I am hungry, I can get food to eat’, is related to the personal resilience factor; in the English version, however, it is classified in the relational resilience subscale. Similarly, items 9, 10, and 12 of the English version are related to relational resilience; however, they are related to both relational and personal factors in this study.

#### Internal reliability/consistency

Cronbach’s alpha correlation coefficient was used to test the internal reliability of the revised resilience questionnaire used in our study. The internal correlation coefficient of the first factor with the 9-items was 0.79, and the second factor with the 7-items was 0.83. The total of 16 items had a correlation = 0.86, indicating the internal correlation of the tool and its two factors.

#### Content and face validity

The International Resilience Project at the RRC created the measures. They investigated 14 communities in 11 different countries that prepared statements for the measures and had then reviewed by local advisors and experts in cross-cultural resilience. The teams checked the sensitivity of the product as a measure of resilience in a social-ecological context. Various experts in the field in different countries have verified this fact ever since (Ungar *et al.*, 2008; Daigneault *et al*., 2013). According to our research, the content and face validity of this test is acceptable in Iran.

### Qualitative procedure and analysis

Using qualitative methods, semi-structural interviews were conducted with all medical staff (*n* = 403). They asked about their family, personal, relational, and organizational problems, which could affect their resilience during the COVID-19 epidemic. A practiced psychologist interviewed each participant separately for 10 min in a private room in their workplace in the hospital to investigate their problems during the COVID-19 epidemic. The main questions were prepared according to the qualitative research by Ungar *et al.* (Ungar and Liebenberg, 2011) and modified for the hospital conditions during the pandemic. The main questions included the following:

– What personal problems have affected your resilience as a medical worker during the COVID-19 epidemic?
– What family and relational problems have affected your resilience as a medical worker during the pandemic?
– What organizational problems have affected your resilience as a medical worker during the pandemic?

**Figure 1.**
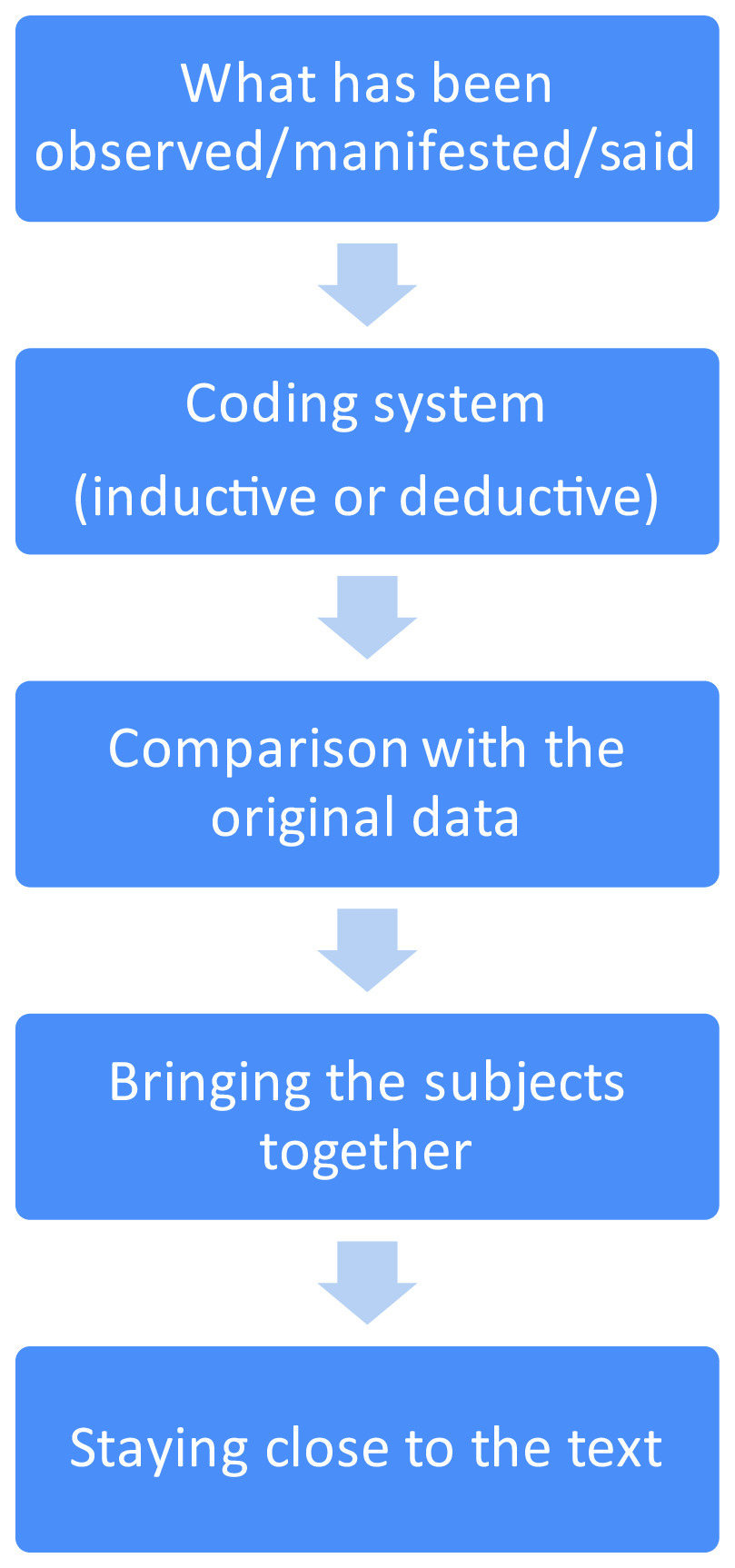
Manifest content analysis process.

The oral interviews were recorded and transcribed by the interviewer. A statistical analyst then used the transcripts for the manifest content analysis, which is defined as describing what is occurring on the surface, what is and literally present, and as staying close to the text (Figure 1). Manifest content analysts deal with the visible data and what is occurring at the surface, which would not require attention to underlying meanings and implications (Kondracki *et al*., 2002; Krippendorff, 2018).

Subsequently, a total of 35 meaning units were identified. After condensation of the units and extraction of the codes, 27 subcategories and finally three categories were defined. The coding systemization was inductive. Tables 5 and 6 show the coding results, categories, and subcategories.

**Table 5.** Code results from the manifest content analysis after condensing the meaning units

1- Not enough resting time/ paid leave	15- Occupational stress
2- Financial problems/ high living expenses	16- Physical illness interfering with work
3- Lack of personal protective equipment	17- Being a contract worker
4- Irregular payments, salary delays	18- Long working hours
5- Discrimination between workers (fully employed versus contract workers)	19- Long working shifts
6- Spouse’s illness	20- Being away from family members and children
7- Educational problems	21- Difficulties in receiving higher education
8- No health insurance	22- Problem in work promotion
9- Administrative negligence	23- Family issues
10- Being overlooked	24- Fear of passing on the infection
11- Insufficient personal facilities	25- Incompatibility of education with the job role
12- Delay in receiving COVID-19 diagnosis kits	26- Physical problems, for example Severe headaches
13- Insufficient workforce	27- Psychological problems, for example stress and anxiety
14- Occupational pressure	

**Table 6.** Categories and subcategories based on the manifest content analysis results

Categories	Subcategories
1- Personal problems	Occupational neuroticism
	Organizational neuroticism
	Physical problems
	Psychological problems
	Mental exhaustion
	Financial difficulties
	Unpredictability and the ambiguous nature of the illness
2- Environmental problems (organization, society)	Organizational discrimination
	Occupational injustice
	Role ambiguity
	Employment insecurity
	Neurotic environment
	Lack of protection equipment
	Lack of personal protection equipments
	Delay in receiving protection equipments
3- Family problems	Emotional distancing
	Family member illness
	Problems and discontent in the marriage
	Missing one’s family
	Fear of getting infected and passing on the infection to family members

The credibility of the results was evaluated through an audit check by five members of the medical staff. Furthermore, an expert panel of two head-nurses reviewed and verified the results. The research team evaluated the dependability of the results by multiple-checking the findings and codes and were in complete agreement over the naming and extraction of codes and categories.

The following are excerpts from the interviews.

– A 29-year-old male emergency resident: ‘I’m finding it very difficult not being able to visit my family for so long. At the same time, I’m worried about catching the virus as I have seen so many Corona patients die during my working shifts’.
– A 27-year-old female registered nurse: ‘I have been self-quarantined for a month. I’m so worried about catching Corona and transmitting the disease to my family. I haven’t visited them since and I’m finding it unbearable’.
– A 29-year-old female pediatrician: ‘I’m working long shifts, yet I’m not even insured and my salary is very small’.
– A 25-year-old female psychologist: ‘The role and duties of a psychologist during the Corona epidemic is not defined and is ambiguous, and there is no supervisor to assign our duties’.
– A 33-year-old male nurse: ‘I’m a contract worker, and I experience discriminatory behavior compared to full-time employees…I’m not compensated for the long working hours…I earn minimum wage, even compared to other contract workers, and it’s not even paid on time’.
– A 26-year-old female registered nurse: ‘We are running out of enough protective equipment such as masks and gloves… We are not paid enough and not on time’.
– A 28-year-old female working at the emergency department: ‘Our working shifts are overwhelming and we have very little paid leaves… I have a long employment record, yet I haven’t been offered a promotion’.
– A 46-year-old male ICU intensivist: ‘We are under an overwhelming pressure due to the nature of the pandemic, long and exhausting working shifts, and having to wear these uncomfortable protective gear all the time… I’m overstressed, I have problems with eating and sleeping, I suffer from nightmares and headaches’.
– A 42-year-old male nurse: ‘I’m not satisfied with my job for many reasons ever since the Corona epidemic… I have a constant feeling of hopelessness and despair, and I suffer from panic attacks…’
– A 35-year-old female emergency worker: ‘The working shifts are exhausting… We are short in staff, and we have to overwork because of that and the huge number of patients… I’m distressed and can’t take care of my children well’.
– A female ICU specialist: ‘The working hours are too long and heavy, especially for us women… We’re more delicate and more easily exhausted under all the protective equipment we have to wear… Long working shift, all the while wearing protective coveralls and N95 masks in a hot environment with low oxygen, has left me fatigued… I’m afraid of removing my mask to drink some water… I’m scared of using the bathroom because I’m afraid of taking off my protective clothes in case of catching the virus… In addition to all of that, we also have to take care of our children and husbands and do the house chores’.
– A 34-year-old female doctor in the Corona department: ‘I’m afraid of dying of Corona… I’m worried about what will happen to my daughter if I die… I’m scared of how devastated she would be’.

### Quantitative procedure and analysis

#### Stepwise regression analysis

The aim of this section was to determine if the demographic and work-related variables can predict the resilience of medical staff or not. First, in each group of medical staff, correlations between the total score of resilience and demographic variables including age, gender, marriage status, number of children, age of children as well as work-related variables such as work experience and continuous workdays were calculated separately. Work experience referred to the number of years participants worked in the current position and continuous workdays mean how many days did they worked continuously from their last leave. This information was retrieved from the hospital record system. As shown in Table 7, in the coronavirus group, marriage status and in the non-coronavirus group, the age and gender of participants as well as their children’s number and age had significant correlations with resilience. But in the emergency group, there were no significant correlations.

**Table 7.** Pearson’s correlations between total resilience, demographic and work-related variables

Groups	Total resilience		
	Coronavirus group	Non-coronavirus group	Emergency group
Marriage status	0.196^*^	−0.078	0.084
Age	−0.049	−0.195^*^	−0.048
Gender	0.072	−0.234^*^	0.054
Education	0.098	0.080	0.104
Number of children	0.049	−0.197^*^	0.022
Age of children	−0.002	−0.266^*^	−0.058
Work experience	−0.088	-0.160	−0.066
Continuous workdays	0.087	-0.105	−0.027

* *P* < 0.05.

Subsequently, stepwise multiple regression was performed on the variables that were correlated significantly with resilience in two groups of medical staff. SPSS excluded variables that their F value was not significant in stepwise regression models. In coronavirus group only marriage status and in non-coronavirus group only age of children was significant and included in the first and only steps of the models. Therefore, it is possible to predict resilience based on these two variables. The summary of the analysis is shown in Table 8.

**Table 8.** Summary of stepwise regression analysis model in coronavirus and non-coronavirus groups

	R	R^2^	F	R Square change	F change	B	β	t
Model 1 (Coronavirus group) Step 1: Marriage	0.196	0.038	4.267^*^	0.038	4.267^*^	3.506	0.195	2.065^*^
Model 2 (Non-coronavirus group) Step 1: Age of children	0.274	0.075	7.879^*^	0.075	7.879^*^	-0.251	-0.274	-2.807^*^

Dependent variable: total resilience.

* *P* < 0.05.

The marital status with a beta of 0.19 can explain about 3.8% of the resilience variance in medical staff who worked in the coronavirus section. The age of children with beta -0.251 is able to explain the 7.5% variance of the resilience in the non-coronavirus group.

#### Resilience means comparison between three groups

The three resilience group means were compared using a one-way analysis of variance (one-way ANOVA). Table 9 shows a significant difference between the personal and relational dimensions and the total score of the three resilience groups (*P* < 0.05). Pairwise comparisons were made using Scheffé’s post hoc test, and the results are shown in Table 10.

**Table 9.** Means, standard deviations, and one-way ANOVA results for resilience scores

		Coronavirus	Non-coronavirus	Emergency	Between groups			Within groups			F	*P*
					SS	df	MS	SS	df	MS		
Personal resilience	Mean	33.60	34.53	32.66	254.88	2	127.44	9101.39	366	24.86	5.13	0.006
	SD	4.85	5.06	5.00								
Relational resilience	Mean	29.18	29.31	27.64	203.26	2	101.63	8273.3	366	22.6	4.5	0.012
	SD	4.38	4.38	5.30								
Total resilience	Mean	62.79	63.85	60.30	741.49	2	380.75	24756.52	366	71.31	5.21	0.006
	SD	8.08	8.37	9.11								

SD: standard deviation.

**Table 10.** The post hoc Scheffe test results

Resilience subscales			Mean differences	*P*-value
Personal	Corona	Non-corona	−0.83	0.421
	Non-corona	Emergency	2.02	0.007
	Corona	Emergency	1.18	0.158
Relational	Corona	Non-corona	−0.078	0.999
	Non-corona	Emergency	1.62	0.028
	Corona	Emergency	1.53	0.044
Total	Corona	Non-corona	-1.61	0.028
	Non-corona	Emergency	3.55	0.007
	Corona	Emergency	2.49	0.09

Scheffé’s test showed a significant difference in personal resilience between the non-coronavirus and emergency groups. The test further showed a significant difference in relational resilience between the non-coronavirus and emergency groups and between coronavirus and emergency groups. Also, the total resilience test showed significant differences between coronavirus and non-coronavirus groups and between non-coronavirus and emergency groups (*P* < 0.05). The resilience means of each group shown in Table 9 indicates the following: the personal resilience in the non-coronavirus is higher than the emergency (M = 34.53 > M = 32.66), the relational resilience in the non-coronavirus and coronavirus is higher than the emergency group (M = 29.31, M = 29.18 > M = 27.64), and the total resilience in the non-coronavirus group is higher than the coronavirus and emergency groups (M = 63.85 > M = 62.79, M = 60.30).

## Discussion

The surprisingly sudden and extreme outbreak of COVID-19 occurred suddenly and severely and the medical staff in the hospitals faced great work stress, and they are prone to be in a state of high mental problems. The medical staff in direct contact with COVID-19 patients has a greater risk of infection and will consequently suffer greater mental stress and is more vulnerable to negative emotions lowering their resilience. If there are not high levels of resilience among medical staff, they not only cannot recover from suffering stress but they also may gradually accumulate negative emotions, which may even lead to developing psychological disorders. The present research used mixed methods to follow four main goals discussed below.

### The psychometric properties of the ARM-R

The first goal was evaluating the psychometric properties of the Persian version of the 17-item ARM-R for the first time. The measure underwent forward translation and backward translation, and the final version was pilot-tested and revised based on the feedback. The researchers could therefore ensure the accuracy of the translations in retaining the meaning of the original items and the clarity of the translated questionnaire. Analytical methods were used to verify the internal reliability/consistency, content validity, and face validity of the Persian version of the measure. The construct validity of the test was verified using exploratory factor analysis. The two-factor structure of the questionnaire was confirmed by eliminating one item. In the Persian version, item 6 (‘If I am hungry, I can get food to eat’) is related to the personal resilience factor; however, this item is a subscale of relational resilience in the English questionnaire.

### The qualitative study of the problems affecting the medical staff’s resilience

The other goal of the present research was investigating the personal, relational, social, relational, and organizational issues facing the medical staff members during the COVID-19 outbreak using qualitative research. The research was carried out using a semi-structural interview. The content of the interviews was analyzed using manifest content analysis, and the results were divided into 27 subcategories and three main categories. The main categories included personal problems (eg, psychological issues), organizational problems (eg, discrimination at work), and family problems (eg, homesickness/missing family members). Other researches have indicated similar issues during the COVID-19 pandemic, including lack of personal protective equipment (PPE) (Chen *et al*., 2020; Huang *et al*., 2020), psychological issues (Chen *et al*., 2020; Huang *et al*., 2020; Liang *et al*., 2020; Lu *et al.*, 2020; Petzold *et al*., 2020; Xiao *et al*., 2020), physical problems (Chen *et al*., 2020; Huang *et al*., 2020), fear of infection of oneself and infecting one’s family (Chen *et al.*, 2020; Huang *et al.*, 2020; Montemurro, 2020; The Lancet, 2020), organizational discrimination (Montemurro, 2020), occupational stressors (Zhang *et al.*, 2019), stressful workplace (Chen *et al*., 2020), role ambiguity, and being away from home (Chen *et al*., 2020) in the medical staff. For instance, the following problems observed by Shanafelt *et al*. are very similar to our findings in Iran: unavailability of COVID-19 tests, fear of virus infection at the workplace, lack of trust in the institution’s support in case of infection, limited childcare during the lockdown, and insufficient knowledge about the disease/infection. Although different studies separately addressed the problems of the medical staff during the pandemic, none of them, like the present study, divided them into semantic categories. This classification helps make more accurate future decisions and better intervention plans.

### Demographic predictors of resilience

The results of regression in this research show that a small portion of resilience variance is related to participants’ marital status and children’s age. These results are in line with Southwick *et al.* (2014) research that discussed when we measure numerous distinct predictors, no single predictor accounts for considerable variance. That is, no single demographic, psychological, or biological characteristic has been demonstrated to significantly predict or enhance resilience by more than a small amount.

The results of the stepwise regression in the present study showed that the marital status was not predictive of resilience changes in the non-coronavirus and emergency groups, but it accounts for 3.8% of resilience changes in the coronavirus group. Marriage had a strong predictive effect for well-being in some populations but a non-significant effect in others, according to the findings of some studies. As indicated by Karaşar and Canli (2020), Turkish people’s psychological resilience was not predicted by marriage at the time of the pandemic. However, unmarried status was associated with a high level of burnout risk, according to the findings of a meta-analysis (Kesarwani *et al*., 2020). Furthermore, many studies have suggested that social support is a crucial factor predicting resilience throughout the lifespan (Horton and Wallander, 2001; Ozbay *et al*., 2008; Martínez-Martí and Ruch, 2017). In the light of existing literature, it can be said that being married in participants who worked in coronavirus section boosted their resilience since it was related to having greater social support (De Silva *et al.*, 2005; Harandi *et al*., 2017), meaning for life, and emotional support (Kim and Mckenry, 2002).

In addition, the results of the present research revealed that the children’s age of medical personnel who were classified as non-coronavirus negatively predicted their resilience; however, this element was not predictive in the other two groups. Because there is not much research concerning the association between demographic characteristics of children and their parents’ resilience, studies conducted on parental distress can be considered to interpret these findings. According to the results of such studies, a child’s age is either adversely associated with parental anxiety or has no association with it (Williford *et al*., 2007; McStay *et al.*, 2014). Nevertheless, a meta-analysis (Barroso *et al.*, 2018) concluded that, due to the inconsistent results, further research is required to determine how child age influences parental stress. Overall, during the COVID-19 pandemic, different variables predicted resilience in different groups of medical workers who had diverse jobs and situations. More investigation is necessary to investigate the association between demographic characteristics and resilience in healthcare practitioners with various conditions; therefore, interpretation of the results mentioned above should be performed cautiously.

### Comparing the resilience in different groups of staff members (coronavirus, non-coronavirus, and emergency)

The other goal was comparing three groups of medical staff: coronavirus group consisted of the medical staff in direct contact with COVID-19 patients; emergency group consisted of the medical staff working in the emergency department who deals with both COVID and non-COVID patients; and non-coronavirus group, consisted of the staff who had no contact with COVID-19 patients.

The results showed the total resilience of the non-coronavirus group was greater than the coronavirus and emergency groups. Also, the relational resilience of the coronavirus and non-coronavirus groups was greater than the emergency group. The personal resilience in the non-coronavirus group was greater than the emergency group. Altogether, the emergency group had a lower resilience in all dimensions compared to the other two groups.

Because sudden outbreak of the novel COVID-19 and the subsequent pandemic blindsided the medical staff and facilities, direct contact with COVID-19 patients, heavy workloads, long working hours, and the increasing number of patients made the coronavirus and emergency groups extremely more vulnerable to psychological traumas (eg, from watching patients suffer or die) and stresses (eg, getting infected and transmitting the infection to their families). These overwhelming and stressful factors have all affected the resilience scores of the medical staff in contact with COVID-19 patients. On the other hand, the total resilience of the emergency group was even lower than that of the coronavirus group. The probable reason is that the emergency ward was less protected against COVID-19 infection. The COVID-specific wards had more protection and isolation to minimize the risk of infection, and the medical staff had little contact with the patients and no contact at all with those accompanying the patients. Also, many patients in the emergency center had symptoms of the COVID-19 but had not been tested for the disease, and their unknown condition made these patients and those accompanying them extremely stressed. This further added to the pressure experienced by the emergency medical staff. On the other hand, the patients entering the COVID-19 wards were assured of their conditions, had some level of acceptance, and had received primary care. Therefore, the COVID-19 medical staff experienced much less pressure.

Researchers have suggested that the resilience of the medical staff might be the result of complex interactions between individual and environmental (workplace, organization) factors and their preliminary attempts to resolve the complexity of the stressors (Lowe, 2013; Tubbert, 2016). Early studies on resilience focused on individual traits or points of strength, which helped individuals to cope with adverse events. However, current researches concentrate on the important role of the contribution of systems forces or support systems that are hugely beneficial for individuals in their counteraction with environmental stressors (Herrman *et al.*, 2011). Therefore, a resourceful environment can have an essential effect on preserving resilience in stressful situations (Ungar, 2019). Studies have shown supportive working environments (Lowe, 2013) and occupational satisfaction (Matos *et al.*, 2010) can preserve resilience even in difficult situations. As was mentioned in the qualitative analysis, however, this study reported numerous environmental and organizational problems and a lack of sufficient support of the medical staff. The sudden outbreak of COVID-19 and the subsequent issues required immediate actions and provision of extra supplies and facilities, especially for the frontline COVID-19 workers. Yet, problems such as lack of PPE, long working shifts, delayed payments and no pay raise for the staff, insurance issues, organizational discrimination, occupational injustice, role ambiguity, lack of job security, and a neurotic workplace led to the absence of a resourceful environment. This factor further contributed to the low scores of the coronavirus and emergency medical staff in the resilience test.

The results of the present study were in accordance with the findings of Matos study, which showed the total resilience scores of the medical workers who were in direct contact with COVID-19 patients in radiology wards were lower than medical students (Matos *et al.*, 2010). Maunder (Maunder *et al.*, 2003) also suggested that the medical professionals working on the frontline of COVID-19 experienced high levels of psychological stress and losses and had to face numerous challenges. These psychological challenges, while lowering resilience, can also trigger symptoms of anxiety and depression. A study by Lu (Lu *et al.*, 2020) showed that compared to non-clinical workers, frontline medical staff in direct contact with COVID-19 patients (including workers of respiratory, emergency, and infectious diseases departments and ICUs) had higher scores on the fear scale, HAMA and HAMD. They experienced fear 1.4 times more than other medical workers and were twice as likely to show symptoms of depression and anxiety.

Moreover, personal and relational subscales of the resilience of the emergency staff were also poorer than other groups. Caregiver/relational resilience involves characteristics of the mutual relationships between a primary caregiver, a partner, or a family. Personal resilience involves intrapersonal and interpersonal factors. The two resilience subtypes are closely connected because they both rely on individuals’ social ecologies for the reinforcement of their respected resilience (manual test). Several researches have indicated that supportive relations and care, such as positive personal relations, effective work relations, and a strong relationship with one’s family are essential factors to improve resilience at times of difficulty and crises (Ablett and Jones, 2007; Gillespie *et al.*, 2007; Jensen *et al.*, 2008). Studies by Mealer (Mealer *et al.*, 2012) showed that nurses with high levels of resilience reported a positive, supportive social network consisting of intimate personal friendships, close relationships with the family, and strong relations with the other medical staff members as one of the essential contributors to their resilience. Supportive relationships with one’s social network can provide great opportunities to process a negative experience by verbalizing and explaining the experience and its associated feelings (Grace and VanHeuvelen, 2019).

## Conclusion

A factor analysis has confirmed the psychometric validity of the Persian version of the ARM-R that was used to study the resilience of medical staff during the peak of the coronavirus pandemic. According to qualitative results, the overall resilience issues among medical staff might be attributable to personal, organizational, and family problems. Some problems such as lack of PPE, discrimination among workers, being overlooked, an insufficient workforce, long working shifts, fear of passing on the infection, financial problems have been reported in the present study. Quantitative results also demonstrated that in some specific medical staff groups, there is a possibility that the ages of medical staff’s children adversely affect their resilience, while their marital status might increase it. The results also showed that the emergency group of medical staff had lower scores in both personal and relational resilience subcategories than the other two groups during the COVID-19. This may be due to the heavy workload of the emergency staff and the infectious nature of the virus, both of which forced them to spend more time at the hospital and away from their families and to live in separate spaces to prevent transmitting the infection. Consequently, they found much less time to socialize with their coworkers than the coronavirus group.

Overall, the accumulation of the stress and psychological pressure of the unknown nature of COVID-19 in addition to the personal, organizational, and family issues overwhelmed the medical staff and minimized their resilience.

### Study limitations and suggestions

The present research faced some limitations. This study was conducted in only one hospital, and it is needed to study more hospitals to generalize the results. Moreover, the present study had a cross-sectional design; for better observing the changes over time, the resilience of the medical staff needs to be investigated further by longitudinal research. Besides, the effect of demographic factors such as marital status and number of children on the resilience of medical staff with various work responsibilities and conditions should be further researched. Finally, future studies should be conducted to determine whether the resilience of the medical staff is improved through positive organizational, personal, and relational changes.

### Implications

Determining the psychometric properties of ARM-R could help researchers and practitioners in primary health care to use a short and valid questionnaire to assess resilience. Detecting low levels of resilience, both in patients and in medical practitioners, and taking timely action to improve it can prevent further consequences. Besides, it is of the highest importance for the hospital administrators to familiarize themselves with the devastative psychological outcomes of unexpected crises like the COVID-19 pandemic on the medical staff, especially the frontline workers who are in direct contact with infected patients, and minimize the pressure and improve their resilience by every possible measure. Strategies such as flexible working hours, providing PPE for all of the staff, setting up lounges and resting spaces, removing role ambiguity by providing a full and clear explanation of tasks and duties, and providing psychological services to deal with the coronavirus situation and increase the medical staff resilience can be of great positive effect in times of crisis.
